# New insights into the limited thermotolerance of anhydrobiotic tardigrades

**DOI:** 10.1080/19420889.2020.1812865

**Published:** 2020-09-09

**Authors:** Ricardo Cardoso Neves, Robyn M. Stuart, Nadja Møbjerg

**Affiliations:** a Department of Biology, August Krogh Building, University of Copenhagen, Copenhagen, Denmark; b Data Science Laboratory, Department of Mathematical Sciences, University of Copenhagen, Copenhagen, Denmark

**Keywords:** Cryptobiosis, desiccation, Ecdysozoa, extreme environments, global warming, high temperatures, meiofauna, Tardigrada

## Abstract

The recent discovery of an upper limit in the tolerance of an extremotolerant tardigrade to high temperatures is astounding. Although these microinvertebrates are able to endure severe environmental conditions, including desiccation, freezing and high levels of radiation, high temperatures seem to be an Achilles’ heel for active tardigrades. Moreover, exposure-time appears to be a limiting factor for the heat stress tolerance of the otherwise highly resilient desiccated (anhydrobiotic) tardigrades. Indeed, the survival rate of desiccated tardigrades exposed to high temperatures for 24 hours is significantly lower than for exposures of only 1 hour. Here, we investigate the effect of 1 week of high temperature exposures on desiccated tardigrades with the aim of elucidating whether exposure-times longer than 24 hours decrease survival even further. From our analyses we estimate a significant decrease in the 50% mortality temperature from 63ºC to 56ºC for *Ramazzottius varieornatus* exposed to high temperatures in the desiccated tun state for 24 hours and 1 week, respectively. This negative correlation between exposure-time and tolerance to high temperatures probably results from the interference of intracellular temperature with the homeostasis of macromolecules. We hypothesize that high temperatures denature molecules that play a vital role in sustaining and protecting the anhydrobiotic state.

## Introduction

Tardigrades are microscopic, aquatic invertebrates with a worldwide distribution [1] and the ability to tolerate extreme environmental conditions[2]. Commonly known as water bears, the members of phylum Tardigrada require a film of water surrounding the body to be active. However, when environmental conditions are hostile, many tardigrades have the ability to enter cryptobiosis – a reversible ametabolic state of life. This physiological condition is common especially among limno-terrestrial species, i.e., species living in terrestrial microhabitats with temporary access to freshwater[2]. Different strategies of cryptobiosis have been described based on the environmental cues they are induced by: anhydrobiosis (desiccation), anoxybiosis (oxygen depletion), chemobiosis (high toxicant concentrations), cryobiosis (extremely low temperatures) and osmobiosis (high solute concentration) [2–4]. Anhydrobiosis – the best-studied form of cryptobiosis – refers to the ability to survive evaporative water loss [5–8]. In order to endure desiccation, tardigrades form a quiescent, barrel-shaped “tun” contracting the body longitudinally and retracting their head and legs [9,10].

The ability to withstand complete desiccation provides tardigrades with an extraordinary tolerance toward extreme conditions, including exposure to space[11]. Tardigrades likely express an assortment of bioprotectants that protect cell membranes, DNA and proteins against damage, while the water evaporates from their bodies and desiccation ensues. Suggested bioprotectants include trehalose that can replace water during dehydration and stabilize cellular structure through vitrification [8,12,13]. Other proposed molecular adaptations include heat shock proteins (HSPs) and late embryogenesis abundant (LEA) proteins that act as molecular shields or chaperones and/or participate in the repair process after desiccation [14,15], as well as the recently discovered “tardigrade-unique proteins” [16,17]. In addition, it has been proposed that muscle protein filaments may be essential for sustaining the structural integrity of the anhydrobiotic tun state [9,18]. Post-cryptobiotic survival of tardigrades, furthermore, seems to be related to a number of genes encoding proteins involved in antioxidant defense and possibly transcription-coupled DNA repair [19,20].

The mechanisms that allow tardigrades to survive desiccation and consecutively tolerate other severe environmental conditions are, however, insufficient to protect them from prolonged periods of high temperature exposure[18]. Desiccated tardigrades have been reported since the mid-19^th^ century to tolerate exposures to heat-shocks (>100ºC) for a short period [21–23], but our recent study reveals their inability to endure high temperatures for periods exceeding 1 hour[18]. Specifically, our thermotolerance experiments on the limno-terrestrial extremotolerant tardigrade *Ramazzottius varieornatus* showed that the otherwise highly resistant desiccated tun state is strongly affected by the length of time in which it is exposed to high temperatures. Indeed, the estimated median temperature required to achieve 50% mortality following 1 hour exposures was 82.7ºC, while it significantly decreased to 63.1ºC following 24 hour exposures. These observations indicate that high temperatures destabilize and denature molecules essential for the survival of desiccated and ametabolic tardigrades. Exposure-time is clearly a limiting factor giving the desiccated tardigrades a restricted window of high temperature tolerance[18].

The discovery of a negative correlation between exposure-time and tolerance to high temperatures of the otherwise highly resistant *R. varieornatus* tuns raises several important questions. It is, for instance, important to understand if exposure-times longer than those tested so far increase the vulnerability of the anyhdrobiotic tardigrades. To address this question, we investigate the effect of 1 week temperature exposures on desiccated *R. varieornatus*. Our results are compared and discussed with respect to our previously reported data on the effect of high temperature on the anhydrobiotic tuns of *R. varieornatus*[18].

## Methods

### Sampling and pre-experimental conditioning of specimens

Specimens of the herbi-/bacterivorous *Ramazzottius varieornatus* Bertolani and Kinchin, 1993 (Eutardigrada, Ramazzottiidae) used in this study were obtained from a single sediment sample collected in February 2018 from a roof gutter in Nivå, Denmark (55°56.685′N, 12° 29.775′E). This urban microenvironment is characterized by extreme conditions as it freezes during winter and frequently dries out during summer time. The sediment sample was frozen under wet conditions, stored at -20°C and eventually transferred to -80°C until January 2020. Before starting the experiments the sample was thawed, diluted in ultrapure water (Millipore Milli-Q® Reference, Merck, Darmstadt, Germany) and acclimated to 5°C. As the sample contained not only sediment but also moss leaves, plant litter, etc., all specimens used in the experiments were also well-fed. The sample was recurrently examined for tardigrades between January and March 2020 with the aid of a stereomicroscope.

### Experimental procedure to assess thermotolerance of anhydrobiotic tuns

Adult (large) specimens of *Ramazzottius varieornatus* showing pronounced locomotor activity were collected from the sediment sample and transferred to glass dishes using a Pasteur pipette, at room temperature (RT; i.e. 23–25°C). The collected specimens were randomly pooled into groups of ca. 20 tardigrades and each group was subsequently transferred in a small volume of ultrapure water onto a piece of filter paper (ca.1 cm^2^) placed inside a glass dish. Due to the evaporation of water from the filter paper the tardigrades then underwent desiccation, while entering the tun state. The desiccation process was monitored under a stereomicroscope for 1 hour at room temperature ensuring that all specimens had transitioned into a tun. The glass dishes containing the desiccated tuns on filter paper were then placed in a Sicco mini-vitrum desiccator with silica (relative humidity ranged between 34–36% and temperature between 22–23°C) for 1 hour. Afterward, the filter papers with tuns were transferred into 0.2 ml PCR tubes and incubated in a C1000 Touch thermal cycler (Bio-Rad, Hercules, CA) at temperatures of 40, 50, 55, 60 or 65ºC for 1 week. Five filter papers, each with ca. 20 tuns, were used for each temperature exposure, i.e. a total of approx. 500 specimens were used for these high temperature exposures. Subsequently to temperature exposure, the PCR tubes with tardigrades were kept at RT for 1 hour. Finally, the tardigrades were rehydrated by placing the filter paper holding the tuns in an embryo dish with 2 ml of ultrapure water, and kept for 1 hour at RT before being transferred to 5ºC. In addition, five groups of ca. 20 tardigrades (i.e. ca. 100 specimens in total) were desiccated on filter paper, transferred into 0.2 ml PCR tubes, and then kept inside the desiccator chamber (ca. 23ºC) for 1 week as controls. The controls were then kept for 1 hour at RT, rehydrated with 2 ml of ultrapure water for another hour at RT and, finally, transferred to 5ºC. Moreover, selected data obtained from a study we published recently (see Neves et al. 2020 [18]) were also considered in the analyses performed here. In this previous study, desiccated specimens of *Ramazzottius varieornatus* were exposed to temperatures of 40, 50, 60, 65 or 70ºC for 24 hours following a methodological setup similar to the one described above. Data from the 1 week exposures as well as the previous 24 hour exposures are provided in Table 1.

**Table 1.** Percent active *Ramazzottius varieornatus* following exposure of desiccated tun state to temperatures between 23°C and 70°C. The tuns were pooled into 5 groups of ca. 20 specimens (i.e. ca. 100 tuns per temperature) and exposed to high temperatures for 1 week or 24 hours (the latter data are from Neves et al. 2020 [18]). Scoring of activity was performed on fully hydrated tardigrades, 2 hours, 24 hours and 48 hours after heat shock. Data are presented as mean±se percent active tardigrades.

Temperature → Exposure time ↓	RT* Ctrl**	40ºC	50ºC	55ºC	60ºC	65ºC	70ºC
1 week	**2 h**: 99.0 ± 1.0 **24 h**: 99.1 ± 0.9 **48 h**: 96.3 ± 1.7	**2 h**: 96.3 ± 1.7 **24 h**: 97.2 ± 1.1 **48 h**: 98.2 ± 1.8	**2 h**: 89.8 ± 3.6 **24 h**: 92.6 ± 3.1 **48 h**: 90.7 ± 3.3	**2 h**: 55.6 ± 5.2 **24 h**: 81.7 ± 3.3 **48 h**: 86.4 ± 3.5	**2 h**: 0.0 ± 0.0 **24 h**: 1.9 ± 1.2 **48 h**: 0.0 ± 0.0	**2 h**: 0.0 ± 0.0 **24 h**: 0.0 ± 0.0 **48 h**: 0.0 ± 0.0	
24 hours***	**2 h**: 97.0 ± 2.0 **24 h**: 97.0 ± 2.0 **48 h**: 97.0 ± 1.2	**2 h**: 97.9 ± 1.3 **24 h**: 99.0 ± 1.0 **48 h**: 98.1 ± 1.2	**2 h**: 96.1 ± 1.8 **24 h**: 94.1 ± 1.8 **48 h**: 91.1 ± 1.9		**2 h**: 93.9 ± 1.9 **24 h**: 92.9 ± 2.5 **48 h**: 92.9 ± 2.5	**2 h**: 8.0 ± 6.8 **24 h**: 27.7 ± 10.0 **48 h**: 37.6 ± 13.5	**2 h**: 0.0 ± 0.0 **24 h**: 0.0 ± 0.0 **48 h**: 0.0 ± 0.0

*RT = ca. 23ºC **Ctrl = control ***Data from Neves et al. 2020 [18]

### Assessment of tardigrade activity

Following temperature exposures, the tardigrades were monitored under a stereomicroscope for a period of 2 days with activity checks at 2, 24 and 48 hours after the heat shock. The specimens were considered active, and alive, if clear movements of legs and/or main body were observed or when they were reactive to a gentle tactile stimulus with the help of a needle. On the contrary, specimens were considered inactive if they were not showing spontaneous movements of the legs and/or main body, nor reacting to a tactile stimulus. Since it was impossible to ensure that inactive specimens were unequivocally dead, the 48 hour assessment of activity may represent a slight underestimation of survival rates.

### Data analyses and statistics

**Figure 1.**
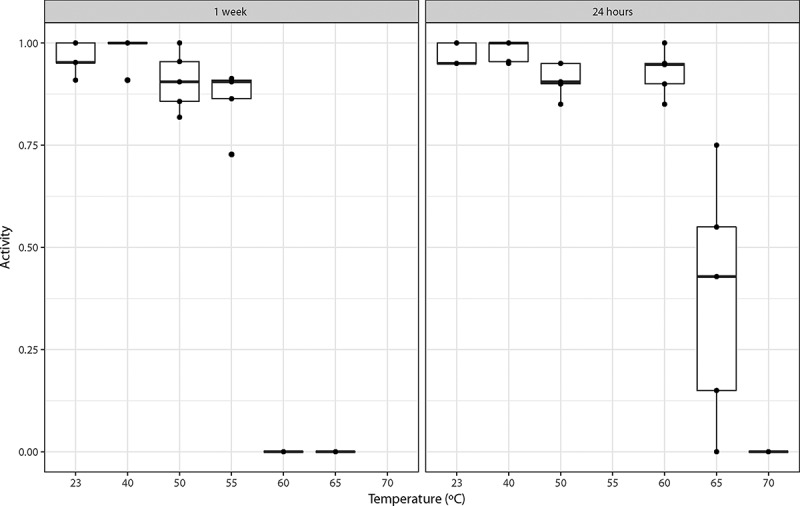
Tardigrade activity (proportion of active animals) 48 hours after exposure of desiccated tuns to high temperatures for 1 week (this study) and 24 hours (from Neves et al. 2000 [18]), respectively. Tardigrades were pooled into 5 groups each containing ca. 20 specimens. Observed data points (•) representing the proportion of active tardigrades in each group are presented together with medians (horizontal lines), interquartile ranges (boxes), and 1.5*interquartile ranges (whiskers).

**Figure 2.**
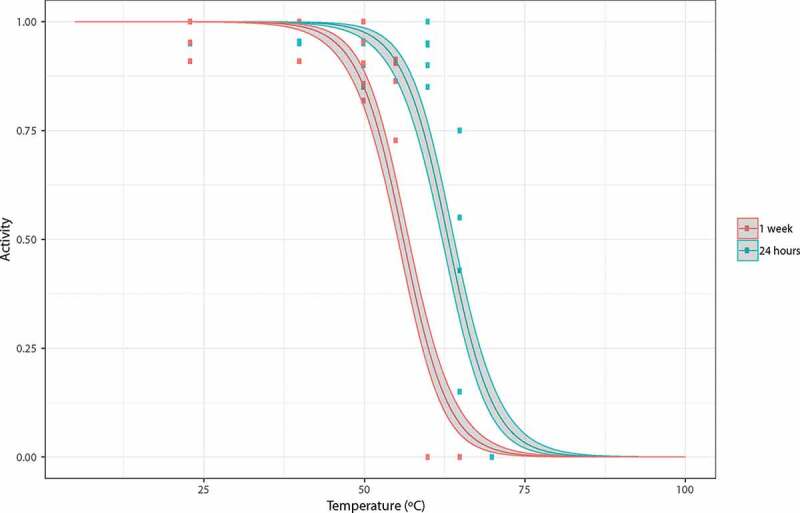
Logistic model of tardigrade activity following exposure to high temperatures of the desiccated tun state. Observed data points (▪) representing the proportion of active tardigrades in each group are presented together with the model estimate and 95% prediction intervals.

Tardigrade activity was calculated as the proportion of active tardigrades, i.e. the ratio between the number of active specimens and the total number of specimens in each replicate group at each time point (i.e. 2 h, 24 h and 48 h after heat shock). These proportions were used as data points and non-parametric summary measures (i.e. medians and interquartile ranges) were used to estimate and plot the distribution of tardigrade activity 48 hours after heat shock (Figure 1). In addition, logistic regression modeling was used to test the effects of exposure time on the survival as described below (Figures 1 and 2). The analyses were performed using R^24^ and plots were produced using the ggplot2 package.

In order to test the effect of exposure time on the survival of desiccated specimens exposed to high temperatures, we created two alternative logistic regression models, each with activity after 48 hours as the binary response variable, and each with duration of exposure (24 hours/1 week) as a factor. The difference between the two models is that one treated temperature as a factor, and the other treated temperature as a continuous variable (a type of dose-response model). The former enabled direct comparisons of the activity of tardigrades at particular temperatures; for this model, we could only include the groups that were exposed to 23, 40, 50, 60 or 65ºC, since tuns were exposed to each of these temperatures for both exposure times (24 hours/1 week). The latter was preferable for estimating the median lethal temperature, i.e. the temperature required to achieve 50% mortality, and included all groups from both exposure times. This model expressed the proportion of animals active after 48 hours of rehydration as follows:

$$PropActive48_{i}=\frac{1}{1+exp\left(-\left(\beta_{0}+\beta_{1}temp_{i}+\beta_{2}E_{i}\right)\right)}+e_{i}$$

where $PropActive48_{i}$ is the proportion of animals in replicate *i* which were observed to be active after 48 hours of rehydration; $E_{i}$ is a dummy variable equal to 1 if the specimens in replicate *i* were exposed for 24 hours and 0 if they were exposed for 1 week; $temp_{i}$ is the temperature that specimens in replicate *i* were exposed to; and the error term $e_{i}$ expresses the difference between the model and the data points. The parameters $\beta_{0}$, $\beta_{1}$ and $\beta_{2}$ were estimated in order to minimize the sum of squared differences between the model predictions and the observations (Table 2). These parameters were used to give an estimate of the median lethal temperature, i.e. the temperature required to achieve 50% mortality: this was given by $-\beta_{0}/\beta_{1}$for the specimens that were desiccated for 1 week and $-(\beta_{0}+\beta_{2})/\beta_{1}$ for the specimens that were desiccated for 24 hours. We validated both models and tested the significance of all effects using approximate likelihood ratio tests.

**Table 2.** Logistic modeling of tardigrade activity following exposure of tun state to high temperatures. Parameter estimates for the logistic regression model with standard errors provided in brackets (compare to Fig. 2).

$\beta_{0}$ (Intercept)	15.860 (1.04)***
$\beta_{1}$ (coefficient of temperature)	−0.282 (0.02)***
$\beta_{2}$ (coefficient of desiccation)	1.976 (0.22) ***

*** Indicates significance at the p < 0.001 level.

## Results

Our approach to assess temperature tolerance of anhydrobiotic tuns (i.e., desiccated tardigrades) was subdivided into two series: one to test 24 hour exposures (data from Neves et al. 2020 [18]) and one to test 1 week exposures (this study). We estimated the survival of *Ramazzottius varieornatus* following rehydration and after exposure to high temperatures in their desiccated state. Survival estimates are based on the observed proportion of active specimens 48 hours after heat shock. The proportion of active tardigrades 48 hours after heat shock was thus used as the response variable in the modeling and statistical analyses.

In Figure 1 we show the proportion of specimens active 48 hours after heat exposure and subsequent rehydration as a function of exposure temperature and exposure time (i.e., 24 hours or 1 week). Based on this data, we found that the survival of desiccated specimens heated to 60ºC was strongly affected by the length of time that they were exposed: approximately 93% of all specimens exposed for 24 hours were active after 48 hours, while 0% of those exposed for 1 week were active. This difference is clearly highly significant (χ^2^(1) = 173.6, p < 0.01). The logistic regression model using the data for all desiccated specimens found that both exposure time of the desiccated specimens and temperature (taken as a continuous variable) were highly significant (χ^2^(1) = 97.91, p < 0.01 for exposure time; χ^2^(1) = 799.65, p < 0.01 for temperature). Parameter estimates for this model are given in Table 2 with standard errors and significance levels. Based on our data we estimate that the median temperature required to achieve 50% mortality among the specimens that were exposed during 24 hours is 63ºC, while it is 56°C for the specimens that were exposed for 1 week. Model predictions, along with confidence intervals, are shown in Figure 2.

## Discussion

Our results show that the ability of anhydrobiotic tuns of *Ramazzottius varieornatus* to endure high temperatures is strongly affected by exposure time. While the median temperature required to achieve 50% mortality (LD50) in desiccated specimens exposed for 24 hours was 63ºC, this parameter decreased to 56ºC for specimens exposed for 1 week. These observations substantiate the results reported in our previous study, where desiccated *R. varieornatus* tolerated much higher temperatures during 1 hour exposures (LD50 = 82.7ºC) as compared to 24 hour exposures (LD50 = 63.1ºC)[18]. It is also interesting to note that the differences in LD50 between 1 hour exposure and 24 hour exposure (i.e., 82.7 – 63.1 = 19.6ºC) is much higher than the differences in LD50 between 24 hour exposure and 1 week exposure (i.e., 63.1  ̶ 56.1 = 7ºC).

The discrepancy in the thermotolerance between 1 hour, 24 hours and 1 week exposures is most likely related to a time-dependent thermal denaturation of macromolecules. We hypothesize that high temperatures denature molecules that are essential for sustaining the desiccated tun state of *R. varieornatus*. It follows that some of the various bioprotectants proposed to be involved in sustaining the anhydrobiotic tardigrade tuns are most likely sensitive to prolonged exposure to high temperatures. However, the significant difference in the survival rate obtained between short versus longer exposure times raises important questions, including which bioprotectants play a role in shielding tardigrades against high temperatures and whether acclimation can provide a tolerance increase. These questions could be addressed in experiments on acclimation [18] and by identifying differentially expressed genes in acclimated and non-acclimated heat-exposed active and tun state tardigrades. Identification of putative temperature dependent expression profiles would allow a possible identification of proteins involved in tardigrade thermotolerance.

Among the proteins with a putative importance for tardigrade thermotolerance are several heat soluble protein families, i.e. the so-called “tardigrade-unique proteins” as well as the very conserved heat inducible Hsp70 family [8,14,16,24–26]. Hitherto found only in eutardigrades, the former belong to protein families that seemingly act as molecular shields during anhydrobiosis [16,19,25]. Expression analysis in *Richtersius* cf. *coronifer* suggested that the expression of Hsp70 is upregulated in active specimens after heat stress induction[27]. Moreover, the tardigrade *Milnesium inceptum* [28] possesses two small α-crystallin heat shock proteins (MtsHsp17.2 and Mt-sHsp19.5), which under heat stress conditions form large complexes that presumably stabilize the structure of other proteins[26]. Interestingly, the expression of one of these heat shock proteins (Mt-shsp17.2) is upregulated by heat-shock treatment of active specimens, though it is not regulated during anhydrobiosis[26]. Proteins with chaperone activity are also known from other stress-resistant invertebrates (e.g., anostracan crustaceans, bdelloid rotifers) that in a dried state are able to endure short exposures to temperatures above 100ºC[29]. Interestingly, in the monogonont rotifer *Brachionus manjavacas* three families of heat shock proteins, namely Hsp40, Hsp60 and Hsp70, seem to play a role in thermotolerance as evidenced by RNAi-mediated gene suppression in hydrated neonate females[30].

Heat shock proteins such as Hsp70 constitute interesting candidates for cytoprotection and resilience against high temperatures, and should be investigated in future gene expression analysis of tardigrades exposed to high temperatures. This approach has proven to be effective to infer transcriptional resilience to heating exposure and predict thermotolerance in *C. elegans*, a model organism often used for studying the effects of heat stress on animals [31,32]. We propose that similar studies on tardigrades could pave the way for a better understanding of thermotolerance and heat stress recovery in these extremotolerant microinvertebrates.

**Table:** Raw data Heat shock 1 week.

Desiccated tardigrades (tuns): heat shocked for 1 week			2 h check						24 check						48 check						
	Replicate	N	#A+	#A	#A+&A	#I	Proportion active	Activity %	#A+	#A	#A+&A	#I	Proportion active	Activity %	#A+	#A	#A+&A	#I	Proportion active	Activity %	
CTRL (29 Jan – 5 Feb)	*1*	21	21	0	21	0	1.000	100.0	20	1	21	0	1.000	100.0	20	0	20	1	0.952	95.2	
	*2*	22	22	0	22	0	1.000	100.0	22	0	22	0	1.000	100.0	21	1	22	0	1.000	100.0	
	*3*	21	19	1	20	1	0.952	95.2	19	2	21	0	1.000	100.0	21	0	21	0	1.000	100.0	
	*4*	21	21	0	21	0	1.000	100.0	20	1	21	0	1.000	100.0	15	5	20	1	0.952	95.2	
	*5*	22	22	0	22	0	1.000	100.0	18	3	21	1	0.955	95.5	18	2	20	2	0.909	90.9	
	***Median***							***100.0***						***100.0***						***95.2***	
	***SE***							***1.0***						***0.9***						***1.7***	
	***Mean***							***99.0***						***99.1***						***96.3***	
	***SD***							***2.1***						***2.0***						***3.8***	
40ºC (13 Feb – 20 Feb)	*1*	21	20	0	20	1	0.952	95.2	20	0	20	1	0.952	95.2	20	1	21	0	1.000	100.0	
	*2*	21	20	0	20	1	0.952	95.2	21	0	21	0	1.000	100.0	20	1	21	0	1.000	100.0	
	*3*	21	20	1	21	0	1.000	100.0	21	0	21	0	1.000	100.0	20	1	21	0	1.000	100.0	
	*4*	22	20	0	20	2	0.909	90.9	20	1	21	1	0.955	95.5	18	2	20	2	0.909	90.9	
	*5*	21	21	0	21	0	1.000	100.0	19	1	20	1	0.952	95.2	21	0	21	0	1.000	100.0	
	***Median***							***95.2***						***95.5***						***100.0***	
	***SE***							***1.7***						***1.1***						***1.8***	
	***Mean***							***96.3***						***97.2***						***98.2***	
	***SD***							***3.8***						***2.6***						***4.1***	
50ºC (24 Fev – 2 Mar)	*1*	23	23	0	23	0	1.000	100.0	23	0	23	0	1.000	100.0	22	1	23	0	1.000	100.0	
	*2*	22	17	0	17	5	0.773	77.3	16	2	18	4	0.818	81.8	18	0	18	4	0.818	81.8	
	*3*	21	18	1	19	2	0.905	90.5	18	1	19	2	0.905	90.5	19	0	19	2	0.905	90.5	
	*4*	22	19	1	20	2	0.909	90.9	21	0	21	1	0.955	95.5	19	2	21	1	0.955	95.5	
	*5*	21	15	4	19	2	0.905	90.5	17	3	20	1	0.952	95.2	18	0	18	3	0.857	85.7	
	***Median***							***90.5***						***95.2***						***90.5***	
	***SE***							***3.6***						***3.1***						***3.3***	
	***Mean***							***89.8***						***92.6***						***90.7***	
	***SD***							***8.1***						***6.9***						***7.3***	
55ºC (3 Mar – 10 Mar)	*1*	22	0	14	14	8	0.636	63.6	17	2	19	3	0.864	86.4	20	0	20	2	0.909	90.9	
	*2*	22	0	10	10	12	0.455	45.5	12	4	16	6	0.727	72.7	14	2	16	6	0.727	72.7	
	*3*	22	0	10	10	12	0.455	45.5	13	4	17	5	0.773	77.3	16	3	19	3	0.864	86.4	
	*4*	21	0	15	15	6	0.714	71.4	14	3	17	4	0.810	81.0	17	2	19	2	0.905	90.5	
	*5*	23	0	12	12	11	0.522	52.2	17	4	21	2	0.913	91.3	20	1	21	2	0.913	91.3	
	***Median***							***52.2***						***81.0***						***90.5***	
	***SE***							***5.2***						***3.3***						***3.5***	
	***Mean***							***55.6***						***81.7***						***86.4***	
	***SD***							***11.5***						***7.3***						***7.9***	
**60ºC (24 Mar – 31 Mar)**	*1*	21	0	0	0	21	0.000	0.0	0	1	1	20	0.048	4.8	0	0	0	21	0.000	0.0	
	*2*	21	0	0	0	21	0.000	0.0	0	0	0	21	0.000	0.0	0	0	0	21	0.000	0.0	
	*3*	21	0	0	0	21	0.000	0.0	0	1	1	20	0.048	4.8	0	0	0	21	0.000	0.0	
	*4*	21	0	0	0	21	0.000	0.0	0	0	0	21	0.000	0.0	0	0	0	21	0.000	0.0	
	*5*	22	0	0	0	22	0.000	0.0	0	0	0	22	0.000	0.0	0	0	0	22	0.000	0.0	
	***Median***							***0.0***						***0.0***						***0.0***	
	***SE***							***0.0***						***1.2***						***0.0***	
	***Mean***							***0.0***						***1.9***						***0.0***	
	***SD***							***0.0***						***2.6***						***0.0***	
**65ºC (5 Feb – 12 Feb)**	*1*	21	0	0	0	21	0.000	0.0	0	0	0	21	0.000	0.0	0	0	0	21	0.000	0.0	
	*2*	21	0	0	0	21	0.000	0.0	0	0	0	21	0.000	0.0	0	0	0	21	0.000	0.0	
	*3*	21	0	0	0	21	0.000	0.0	0	0	0	21	0.000	0.0	0	0	0	21	0.000	0.0	
	*4*	21	0	0	0	21	0.000	0.0	0	0	0	21	0.000	0.0	0	0	0	21	0.000	0.0	
	*5*	22	0	0	0	22	0.000	0.0	0	0	0	22	0.000	0.0	0	0	0	21	0.000	0.0	
	***Median***							***0.0***						***0.0***						***0.0***	
	***SE***							***0.0***						***0.0***						***0.0***	
	***Mean***							***0.0***						***0.0***						***0.0***	
	***SD***							***0.0***						***0.0***						***0.0***	

## Supplementary Material

Supplemental Material

## Data Availability

Raw data generated for the current publication have been uploaded as supplemental material. Computer codes and the associated datasets analysed during the study are available at GitHub: https://github.com/robynstuart/tardigrades
